# Prediction of Molecular Initiating Events for Adverse Outcome Pathways Using High-Throughput Identification of Chemical Targets

**DOI:** 10.3390/toxics11020189

**Published:** 2023-02-17

**Authors:** Veronica Lizano-Fallas, Ana Carrasco del Amor, Susana Cristobal

**Affiliations:** 1 Department of Biomedical and Clinical Sciences, Cell Biology, Faculty of Medicine, Linköping University, 581 85 Linköping, Sweden; 2 Ikerbasque, Basque Foundation for Sciences, Department of Physiology, Faculty of Medicine, and Nursing, University of the Basque Country (UPV/EHU), 489 40 Leioa, Spain

**Keywords:** molecular initiating event, adverse outcome pathway, chemical target, proteome integral solubility alteration assay, thermal proteome profiling, predictive toxicology, multi-criteria decision-making analysis, analytic hierarchy process, TCDD

## Abstract

The impact of exposure to multiple chemicals raises concerns for human and environmental health. The adverse outcome pathway method offers a framework to support mechanism-based assessment in environmental health starting by describing which mechanisms are triggered upon interaction with different stressors. The identification of the molecular initiating event and the molecular interaction between a chemical and a protein target is still a challenge for the development of adverse outcome pathways. The cellular response to chemical exposure studied with omics could not directly identify the protein targets. However, recent mass spectrometry-based methods are offering a proteome-wide identification of protein targets interacting with s but unrevealing a molecular initiating event from a set of targets is still dependent on available knowledge. Here, we directly coupled the target identification findings from the proteome integral solubility alteration assay with an analytical hierarchy process for the prediction of a prioritized molecular initiating event. We demonstrate the applicability of this combination of methodologies with a test compound (TCDD), and it could be further studied and integrated into AOPs. From the eight protein targets identified by the proteome integral solubility alteration assay after analyzing 2824 human hepatic proteins, the analytical hierarchy process can select the most suitable protein for an AOP. Our combined method solves the missing links between high-throughput target identification and prediction of the molecular initiating event. We anticipate its utility to decipher new molecular initiating events and support more sustainable methodologies to gain time and resources in chemical assessment.

## 1. Introduction

Chemicals are widely used and offer significant benefits to our daily lives. However, the number, diversity, and complexity of substances coming to the market are increasing enormously. Just under the European Union legislative frameworks, there are more than 200,000 chemicals registered [1]. Thousands of new and existing chemicals are required to be evaluated. The risk to human health and the environment of many of these compounds is still unknown. Thus, predictive toxicology based on mechanistic information has become critical [2]. The generic structure of an AOP portrays the linkage between a molecular initiating event (MIE) and an adverse outcome at a biological level of organization relevant to risk assessment, i.e., at the level of organism or population, passing across key events and key event relationships [3,4].

In silico, in chemico, and in vitro data [2,5] have been utilized to predict the MIE that would be used to develop the AOP. It has been recommended batteries of in vitro bioassays, using high throughput technologies, scrutinize rapidly and cost-effectively the interaction of individual chemicals with specific molecular targets or biological pathways whose perturbation could lead to adverse outcomes [6,7]. Digging in omics data from changes in expression from transcriptomics and using gene enrichment analysis to identify the molecular network altered by the chemical treatment has been a common strategy [8] that has often failed to identify the protein target interacting with the chemical at the molecular level [9].

In drug development, the identification of all possible targets of a new drug is essential. Understanding that chemicals can interact with proteins even before being metabolized, the biophysical principle of ligand-induced thermal stabilization of target proteins has been used as a tool to identify protein targets and it is called a thermal shift assay. The cellular thermal shift assay (CETSA) method could, for the first time, scrutinize the soluble proteome for possible protein targets and identify them using a few hundred available antibodies [10]. Based on that finding, the thermal proteome profiling (TPP) method could systematically track all changes in proteome thermal stability upon binding to a compound by mass spectrometry and specifically identify a few true targets among several thousands of proteins [11]. An alternative method, the proteome integral solubility alteration (PISA) improved the analytics resulting in increased throughput [12]. Our lab has been the first to modify TPP and PISA for their application to identify targets of environmental chemicals. First, we observed that the TPP centrifugation speed was not sufficient to eliminate microsome vesicles from the assay. Many hydrophobic chemicals were entrapped in the microsomal lipid core modifying the chemical concentration in the assay. Our modified method, bTPP, solved the problem of eliminating membranous vesicles before the chemical interactions. We demonstrated the robustness of the method identifying targets of novel compounds from marine biodiscovery [13]. Recently, we have also modified the PISA method for the identification of targets of environmental chemicals including endocrine disruptor compounds, and chemical mixtures within the proteome from zebrafish embryos, a common model in toxicology [14]. Even though the PISA methodology offers the prediction of all targets in the studied proteome, it is still required to predict the most relevant target to integrate this knowledge into AOPs.

Any problem where a significant decision is required can be solved by the application of multi-criteria decision-making analysis (MCDM) methods [15]. The decisions can be classified into four main types: choice problem, sorting problem, ranking problem, and description problem. In the case of choice problems, the aim is to select the single best option or reduce the group of alternatives to a subset of equivalent good options. Within sorting problems, the alternatives are grouped into categories. In ranking problems, the options are ordered from best to worst using scores or pairwise comparisons. While in description problems, the aim is to describe the alternatives and their consequences [16]. Aspects to consider for choosing the appropriate methodology are the type of problem to be solved, the desired output quality, and the modeling effort able to input. The analytic hierarchy process (AHP) belongs to a family of decision-making tools for choice and ranking problems and offers the highest output quality requiring a medium modeling effort to perform the analysis, making it suitable for assisting the selection of an MIE from the high-throughput data. Within AHP the best alternative is selected by enumerating key factors for decision-making and assessing the relative value of different decision alternatives, integrating evidence-based data [15,17]. Previously, MCDM techniques have been used for the toxicity prioritization of fine dust sources, ranking chemicals for toxicological impact assessments, and assessing the risk from multi-ingredient dietary supplements, but to the knowledge of the authors, it has never been applied to the field of AOPs before [18,19].

In the present study, we propose to accelerate the prediction of MIEs by the combination of high-throughput identification of chemical targets by the PISA assay with AHP. The predicted MIEs could be further studied and used to develop new AOPs. We anticipate the potential applications of the AOP framework for chemical risk assessment.

## 2. Materials and Methods

### 2.1. Sample Preparation

Reagents and medium were purchased from Sigma-Aldrich (Sant Louis, MO, USA) unless otherwise noted. PBS was purchased from Trevigen (Gaithersburg, MD, USA). HepG2 cells were grown until 70% confluence in EMEM medium supplemented with 7% fetal bovine serum (ATCC, Manassas, VA, USA), 1675 mM L-glutamine, 85 U/mL penicillin, and 85 µg/mL streptomycin. Cells were harvested and centrifugated at 340× *g* for 4 min at 4 °C. Three washes were made with 30 mL of cold PBS. Resuspension and centrifugation at 340× *g* for 4 min at 4 °C. Washed pellets were snap-frozen in liquid nitrogen and stored at −80 °C until lysis.

### 2.2. Selection of Test Compound and Concentration

The test compound analyzed in this study was 2,3,7,8-tetrachlorodibenzo-p-dioxin (TCDD) (LGC Standards, Wesel, Germany), a persistent organic pollutant and endocrine disruptor compound, and the corresponding highest concentration tested was 25 nM. The rationale for the selection of the test compound was to include a well-known xenobiotic of high relevance for human and environmental health. Therefore, we included TCDD and its concentration from a previous study for in vitro exposures to the human hepatic cell line HepaRG. Concentration selection was based on the translation of external intakes into internal doses in hepatic cells. The external intake estimates from plasma monitored levels or environmental, accidental, and occupational conditions of TCDD were associated with target tissue (liver cells) dosimetry using a generic physiologically based biokinetic model. The highest serum levels observed correspond to an intake of up to 15 ng/kg_bw/d which corresponded to an internal dose in the liver cells of up to 25 nM TCDD [20].

### 2.3. Two Dimensions Proteome Integral Solubility Alteration (2D PISA) Experiments in HepG2 Cells Protein Extracts

HepG2 cells were resuspended in ice-cold PBS supplemented with protease inhibitors (Clontech, Mountain View, CA, USA) and lysed in an ice bath by sonication in cycles of 10 s on/5 s off for 1 min at 6–10 μm amplitude at 25% intensity from an exponential ultrasonic horn of 3 mm in a Soniprep 150 MSE (MSE Ltd., Lower Sydenham, London, UK). The insoluble parts were sedimented by centrifugation at 100,000× *g* for 60 min at 4 °C. Protein concentration was determined by BCA assay [21]. The soluble proteome was used to perform the 2D PISA assay, as described in Gaetani et al. [12] with some modifications. Briefly, the soluble proteome and the studied chemical were incubated for 10 min at 25 °C. The incubation was performed with TCDD at 10 different concentrations. The studied concentrations were 100, 90, 80, 70, 60, 50, 40, 30, 20, and 0%. The highest concentration (100%) corresponds to 25 nM TCDD. The control sample (0%) was incubated in the presence of the vehicle, dimethyl sulfoxide (DMSO), utilized for the solubilization of the compound. Ten specific temperatures were selected for the thermal assay: 37, 42, 46, 49, 51, 53, 55, 58, 62, and 67 °C. These temperatures were selected to ensure that at least 90% of the studied proteins have their melting point within this range [22]. Aliquots containing 10 μg of protein (one for each of the temperatures in the entire range covered in the thermal shift assay) were independently heated at the corresponding temperature for 3 min, followed by 3 min at room temperature. For each concentration, aliquots of all temperature points were pooled and centrifuged at 100,000× *g* for 20 min at 4 °C, to remove the proteins that had an alteration in solubility after the thermal shift assay. Supernatants from intermediate concentrations were combined. The collection of soluble fractions in the supernatants from the three conditions (control—0%, intermediate concentrations, and highest concentration—100%) were processed using a general bottom-up proteomics workflow and the purified peptides were analyzed by label-free nano liquid chromatography-tandem mass spectrometry analysis (nLC-MS/MS) [22]. Three biological replicates were performed for each experiment.

### 2.4. Filter-Aided Sample Preparation (FASP)

The samples were digested following the FASP method. First, the protein samples corresponding to the supernatants after centrifugation were prepared with SDT buffer (2% SDS, 100 mM Tris-HCl, pH 7.6, and 100 mM DTT). To perform FASP, the samples were diluted with 200 μL of 8 M urea in 0.1 M Tris/ HCl, pH 8.5 (UA) in 30 kDa microcon centrifugal filter units. The filter units were centrifuged at 14,000× *g* for 15 min at 20 °C. The concentrated samples were diluted with 200 μL of UA and centrifuged at 14,000× *g* for 15 min at 20 °C. After discharging the flow-through 100 μL of 0.05 M iodoacetamide was added to the filter units, mixed for 1 min at 600 rpm on a thermo-mixer, and incubated statically for 20 min in dark. The solution was drained by spinning the filter units at 14,000 g for 10 min. The filter units were washed three times with 100 μL buffer UA and centrifuged at 14,000× *g* for 15 min. The filter units were washed three times with 100 μL of 50 mM ammonium bicarbonate. Endopeptidase trypsin solution in the ratio 1:100 was prepared with 50 mM ammonium bicarbonate, dispensed, and mixed at 600 rpm in the thermomixer for 1 min. These units were then incubated in a wet chamber at 37 °C for about 16 h to achieve effective trypsinization. After 16 h of incubation, the filter units were transferred into new collection tubes. To recover the digested peptides, the tubes were centrifuged at 14,000× *g* for 10 min. Peptide recovery was completed by rinsing the filters with 50 μL of 0.5 M NaCl and collected by centrifugation. The samples were acidified with 10% formic acid (FA) to achieve a pH between 3 and 2. The desalting process was performed by reverse phase chromatography in C18 top tips using acetonitrile (ACN; 60% *v*/*v*) with FA (0.1% *v*/*v*) for elution, and vacuum dried to be stored at −80 °C till further analysis.

### 2.5. Nano LC-MS/MS Analysis

The desalted peptides were reconstituted with 0.1% FA in ultra-pure milli-Q water and the concentration was measured using a NanoDrop (Thermo Fischer Scientific, Waltham, MA, USA). Peptides were analyzed in a QExactive quadrupole-orbitrap mass spectrometer (Thermo Fischer Scientific). Samples were separated using an EASY nLC 1200 system (Thermo Fischer Scientific) and tryptic peptides were injected into a pre-column (Acclaim PepMap 100 Å, 75 um × 2 cm) and peptide separation was performed using an EASY-Spray C18 reversed-phase nano-LC column (PepMap RSLC C18, 2 um, 100 Å, 75 um × 25 cm). A linear gradient of 6 to 40% buffer B (0.1% FA in ACN) against buffer A (0.1% FA in water) during 78 min and 100% buffer B against buffer A till 100 min, was carried out with a constant flow rate of 300 nL/min. Full scan MS spectra were recorded in the positive mode electrospray ionization with an ion spray voltage power frequency (pf) of 1.9 kV (kV), a radio frequency lens voltage of 60, and a capillary temperature of 275 °C, at a resolution of 30,000 and top 15 intense ions were selected for MS/MS under an isolation width of 1.2 m/z units. The MS/MS scans with higher energy collision dissociation fragmentation at a normalized collision energy of 27% to fragment the ions in the collision-induced dissociation mode.

### 2.6. Peptide and Protein Identification and Quantification

Proteome Discoverer (v2.1, Thermo Fischer Scientific) was used for protein identification and quantification. The MS/MS spectra (. raw files) were searched by Sequest HT against the Human database from Uniprot (UP000005640; 95,959 entries). Cysteine carbamidomethylation was used as static modification and methionine oxidation as a dynamic modification for both identification and quantification. A maximum of 2 tryptic cleavages were allowed, and the precursor and fragment mass tolerance were 10 ppm and 0.02 Da, respectively. Peptides with a false discovery rate (FDR) of less than 0.01 and validation based on q-value were used as identified. The minimum peptide length considered was 6 and the FDR was set to 0.1. Proteins were quantified using the average of the top three peptide MS1 areas, yielding raw protein abundances. Common contaminants like human keratin and bovine trypsin were also included in the database during the searches for minimizing false identifications. The mass spectrometry proteomics data have been deposited to the ProteomeXchange Consortium via the PRIDE [23] partner repository with the dataset identifier PXD033056.

### 2.7. Analysis of 2D PISA Assay

Two dimensions PISA assay measures the protein abundance from 3 biological replicates of 3 conditions (control—0%, intermediate concentrations, and highest concentration—100% of the tested compound). Protein abundances from control and the highest concentration represent, for each protein, the integral of the area under its melting curve within the used temperature interval. If S_m_ is the value for the control condition and S_m_′ is the corresponding value for the highest concentration condition, then the PISA analog of the melting temperature shift (ΔT_m_) is

F_t_ = ΔS_m_ = S_m_′ − S_m_

Protein abundance from intermediate concentrations (S_m_″) represents an integral of the concentration-dependence curve. Similarly, the PISA analog of the compound concentration required to induce half of the ΔT_m_ (C0) is

F_c_ = (S_m_″ − S_m_)/(S_m_′ − S_m_)

For each protein, the abundance was normalized on the average value for the control condition, and then F_t_ and F_c_ were calculated as described. Two-tailed Student’s *t*-test (with equal or unequal variance depending on F-test) was applied to calculate *p*-values. Proteins with *p*-values < 0.05 for both F_t_ and F_c_ were considered protein targets, meaning to be the proteins’ combined solubility alteration with action at a low compound concentration [12]. The data were represented in a scatter plot combining F_t_ and F_c_ *p*-values, to facilitate the visualization of the protein targets.

### 2.8. Protein-Chemical Binding Validation at the Structural Level

The in silico prediction of noncovalent binding by molecular docking and the assessment of differential scanning fluorimetry was used as orthogonal approaches for the protein-chemical binding validation at the structural level. Predicting interactions between proteins and small molecules by molecular docking has been widely used for deciphering biological processes, understanding protein functions, drug development, and exploration of the binding properties of chemicals [24]. In this case, by comparison with a well-known binding interaction, it will be used for confirming the interaction of TCDD and the targets identified by the PISA assay. The bound conformations, the binding affinity for the targets identified in this study, and the aryl hydrocarbon receptor (AHR), a well-known and established human target of TCDD [25], were predicted and compared. The molecular docking was performed using CB-Dock 2, a user-friendly blind docking web server, which predicts binding modes without information about binding sites [24]. Compared to other docking approaches, the blind approach fits well with our purpose since our targets have not been studied as TCDD targets before and we do not have information about their binding sites. The Vina score binding obtained from CB-Dock2 for the top cavity of each protein was used in the comparison. The more negative score, the better the binding affinity.

Besides, one identified protein target was selected to perform the protein-chemical binding validation through the assessment of differential scanning fluorimetry with a nanoDSF device. NanoDSF is based on the changes in the intrinsic tryptophan fluorescence (ITF) resulting from alterations of the 3D structure of proteins, when proteins unfold, as a function of the temperature. Therefore, a melting temperature (Tm) can be determined [26]. Monitoring of the ITF at 330 nm and 350 nm during protein thermal denaturation was carried out in a Prometheus NT.48 instrument from NanoTemper Technologies (Munchen, Germany) with an excitation wavelength of 280 nm. Excitation power was set at 25%. Capillaries were filled with 10 μL of a solution containing the protein and TCDD, placed into the sample holder, and a temperature gradient of 0.5 °C/min from 20 °C to 80 °C was applied. The ratio of the recorded emission intensities (Em350 nm/Em330 nm), which represents the change in tryptophan fluorescence intensity was plotted as a function of the temperature. The fluorescence intensity ratio and its first derivative were calculated with the manufacturer’s software (PR.ThermControl, version 2.3.1 from NanoTemper Technologies). For validating protein-chemical binding, the purified protein was mixed with 3 different concentrations of TCDD. The tested compound concentrations were 5 nM (20%), 15 nM (60%), and 25 nM (100%). The protein’s final concentration was 0.5 mg/mL. Control was performed with purified protein in PBS and DMSO maintaining the corresponding protein concentration as for the TCDD. Three replicates were carried out for each condition. Selection of the protein target for validation was based on the availability on the market (full-length recombinant protein and without GST tag, due to possible interferences with chemical binding) and the number of tryptophan residues (at least 2).

### 2.9. Selection of Protein Target for New Adverse Outcome Pathways

**Figure 1:**
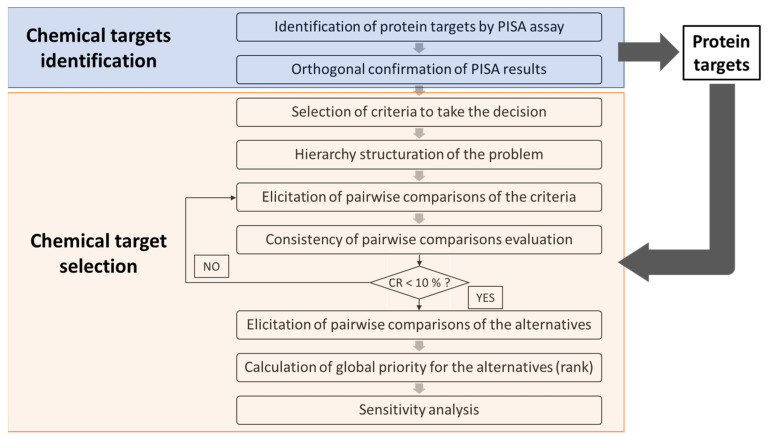
Workflow followed to select from identified protein targets by 2D PISA assay the prioritized one for further studies and for its integration into AOPs. A workflow of the 2D PISA is available in the supplementary information (Figure S1). CR: consistency ratio. Adapted from Yadav and collaborators [29].

From the identified protein targets for TCDD, the multiple-criteria decision-making analysis technique, AHP, allowed the selection of one target as the best-prioritized protein for further studies and integration into AOPs. For a better understanding of the methods, the workflow is shown in Figure 1. The AHP approach arranges the factors considered to decide on a hierarchic structure and relies on three steps [17]. The first one is decomposition. Here, the problem was structured as a hierarchy, where the first level contains the overall goal, i.e., the selection of the prioritized protein which could be used for developing an AOP. The following level corresponds to the criteria which contribute to the goal. These criteria were chosen by the authors based on the requirements and guidelines for developing an AOP [2,5,27]. The third level includes the alternatives (protein targets) to be evaluated in terms of the criteria in the second level.

The second step is the elicitation of pairwise comparison judgments, where a matrix of the relative importance of each criterion over each other was performed using a scale from 1 to 9, according to the expertise of the authors. In this scale, 1 denotes that the two factors contribute equally to the goal, 9 represents the extreme importance of one over another, while 3 indicates slight importance. A numeric scale of 5 represents moderate importance and 7 indicates a very strong relevance of one factor over another. The values 2, 4, 6, and 8 represent the intermediate values between two adjacent judgments.

After calculating the priority vector of the matrix, the consistency of the pairwise comparisons was evaluated through the calculation of the consistency ratio (CR), which involved the following operations:

- Computing the principal eigenvalue (λ_max_) as in Equation (1)
  Aw = λ_max_w (1)
  where A is the priority vector, and w are the eigenvalues of the vector A.
- Computing the consistency index (CI) as in Equation (2)
  (2) $$\mathrm{CI}=\frac{\lambda_{\max}-\;n}{n-1}$$
  where n is the number of criteria
- Calculation of the CR as in Equation (3)
  (3) $$\mathrm{CR}=\frac{\mathrm{CI}}{\mathrm{RI}}$$
  where RI corresponds to the appropriate value of the random consistency indices i.e., the CI expected from a matrix of that order. According to Saaty, the value of RI is 0 up to order 2 while for 3 to 10, the random consistency index values are 0.58, 0.90, 1.12, 1.24, 1.32, 1.41, 1.45, and 1.49, respectively. A consistency ratio of up to 10% is considered acceptable [28].

The third step of AHP is to establish the global priorities of the alternatives. This was done by laying out the local priorities of the alternatives concerning each criterion in a matrix (by pairwise comparison judgments using a scale from 1 to 9). Then, the local priorities are multiplied by the priority of the corresponding criterion and added across each row. The alternative with the highest global priority was selected as the best-prioritized protein for further study and its use for developing an AOP.

Finally, to increase the viability and robustness of the results, a sensitivity analysis was performed. The sensitivity analysis assesses the effects on the final decision after the minor variation in the input. Any slightest change in the current priority can alter the existing global priorities of the alternatives [29]. Here, the criterion with the highest priority was selected and varied from 0.05 to 0.9 in intervals of 0.05 to calculate the global priorities of the alternatives for each interval. If the alternative selected as the best-prioritized protein maintains its position at every interval the result of the AHP method is validated.

## 3. Results

### 3.1. Identification of Protein Targets from a Single Chemical by Applying 2D PISA Assay

We determined the list of proteins interacting with a single chemical, TCDD, used here as a test compound. Applying the 2D PISA method and analyzing by mass spectrometry the proteome-wide alteration in the protein solubility, the specific protein targets from a chemical could be identified. The chemical targets are the proteins that showed an increase or decrease in their thermal stability in a thermal shift assay after the compound was incubated with the soluble proteome of the studied cells. The human hepatic cell line, HepG2, has been selected as the proteome for the target identification. Taking the advantage that the 2D PISA assay can cover the analysis of an extended range of chemical concentrations in a single analytical method, we evaluated 10 different concentrations of the compound, in a dilution series of 10%, starting from 20% of the highest concentration—25 nM (100%). We used three biological replicates per condition and the corresponding control with a vehicle.

**Figure 2:**
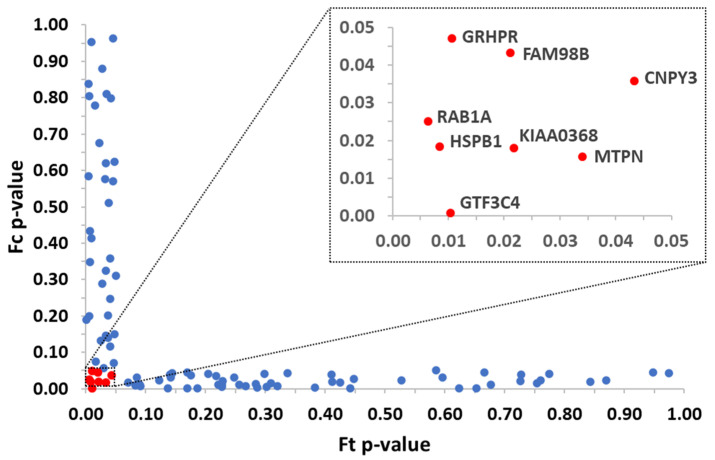
Protein targets were identified by the 2D PISA method for TCDD and the soluble proteome from hepatocytes. The studied concentrations were 100, 90, 80, 70, 60, 50, 40, 30, 20, and 0% (concentrations from 20 to 90% were pooled for the MS/MS analysis and the obtained data were used to calculate F_c_ while data from 100% concentration were used to calculate F_t_). The highest concentration (100%) corresponds to 25 nM TCDD. Identified protein targets are shown in red and labeled. Significant proteins for F_t_ or F_c_ are shown in blue. (For interpretation of the references to color in this figure legend, the reader is referred to the web version of this article).

Out of 2824 proteins identified in the studied proteome, 1475 proteins reproducibly quantified across all three replicates were included in the 2D PISA analysis that yield 8 proteins as targets for TCDD. The protein targets can be depicted in the plot (Figure 2). The protein target general transcription factor 3C polypeptide 4 (GTF3C4) showed the highest solubility alteration at the highest concentration tested of TCDD and action at lower concentrations of TCDD. The other protein targets are heat shock protein beta-1 (HSPB1), ras-related protein Rab-1A (RAB1A), proteasome adapter and scaffold protein ECM29 (KIAA0368), myotrophin (MTPN), protein FAM98B (FAM98B), glyoxylate reductase/hydroxypyruvate reductase (GRHPR), protein canopy homolog 3 (CNPY3).

### 3.2. Orthogonal Protein-Chemical Binding Validation at the Structural Level

Molecular docking was used as one of the protein-chemical binding validation approaches by comparing the affinity score for the targets obtained by PISA assay and AHR, the well-known TCDD target. The obtained affinity score at the top protein pocket or cavity for TCDD and the targets GTF3C4, RAB1A, and HSPB1 were higher than for AHR confirming that TCDD could also bind to them. The other 3 targets showed similar affinity values to the interaction between TCDD and AHR. Another target has a lower affinity. The target KIAA0368 was not included in the comparison because its PDB format was not available (Table 1).

The second protein-chemical binding validation approach used was nanoDSF, since protein melting temperature modification is expected when protein stability changes because of protein-chemical interactions. From the 2D PISA assay, the protein target heat shock protein beta-1 (HSPB1) for TCDD was selected to perform the binding validation with nanoDSF.

**Figure 3:**
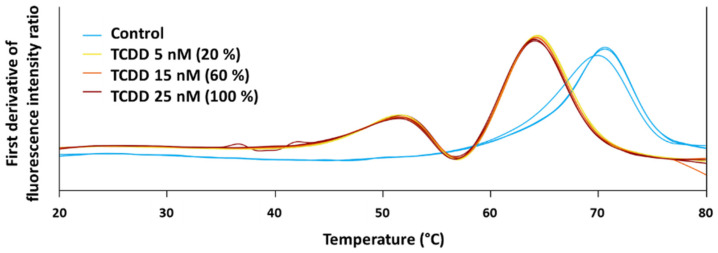
Melting temperatures obtained from nanoDSF protein-chemical binding validation for the interaction of HSPB1 with TCDD at 5 nM (20%) (yellow), 15 nM (60%) (orange), and 25 nM (100%) (red). A control without TCDD was included (light blue). (For interpretation of the references to color in this figure legend, the reader is referred to the web version of this article).

HSPB1 has 6 tryptophan residues, a condition required for this analysis. Purified protein was purchased from Novus Biological as an untagged full-length recombinant protein (NBC1-18364). After performing nanoDSF validation, compared to the controls, a shift in the melting temperature was observed from the first derivative of the ratio of the emission intensities (Em350 nm/Em330 nm) of the interaction between HSPB1 and TCDD at 3 different concentrations, as expected (Figure 3).

### 3.3. Selection of the Prioritized Target for Developing AOPs

We have evaluated an AHP method that includes a semiquantitative analysis for the selection of a prioritized target out of the 8 protein targets identified by the 2D PISA assay that could be used to predict the MIE. The first challenge for the application of AHP was to generate a hierarchical structure of the problem. The first level of the hierarchy was the overall goal of the analysis, i.e., selecting the priority protein target which could be used to develop an AOP. The second level contained 8 criteria that were selected based on the requirements and guidelines for developing an AOP [2,5,27]. These criteria are described in Table 2. The third level included the 8 protein targets identified by the 2D PISA assay as the alternatives to be evaluated in terms of the criteria of the second level.

After defining the 8 criteria for selecting the priority protein target that could be integrated into the development of an AOP, a matrix of pairwise comparison judgments of the criteria was performed by the author’s expertise. Table 3 shows the obtained matrix, the priority vector, and the corresponding λ_max_, CI, RI, and CR. According to the priority vector, the criteria: position in F_t_ (solubility alteration) ranking and position in F_c_ *p*-value ranking obtained the highest weight of relevance for a protein to be used for developing AOPs.

The following criteria in the priority rank were: (i) relevance of reported negative effects on cells/organs/organisms when protein functionality is absent, (ii) relevance of pathways where the protein has participated, (iii) the number of reported negative effects on cells/organs/organisms when protein functionality is absent, and (iv) the number of diseases where the protein is involved. Finally, the lowest weight was occupied by the criterion number of functional and physical protein associations with other protein targets. These are quantitative data available to be retrieved for most of the proteins.

The third step of AHP is to establish the global priorities of the alternatives by pairwise comparison judgments. A priori, a database containing the information of each protein for each criterion was created to perform the comparisons (Table S1). The main sources utilized to retrieve the information for the database are indicated in Table 2. The matrices of pairwise comparison judgments of the alternatives were performed by the authors, and the corresponding local priority vectors, λ_max_, CI, RI, and CR are shown in Table S2. The local priorities were multiplied by the priority of each criterion. The obtained values were added to derive the global priority of each protein (Table 4). Protein HSPB1 reached the highest global priority (0.414), turning it into the alternative selected by the AHP strategy as the highest prioritized target for further being deeply studied and coupled in the development of AOPs.

Besides, AHP results were validated through a sensitivity analysis. Due to the criterion position in F_t_ (solubility alteration) ranking having the maximum priority it was used as input to assess the minor variation on the final decision, varying it from 0.05 to 0.9 in intervals of 0.05 to calculate the global priorities of the alternatives for each interval. Table 5 shows that a minor variation occurs in the ranking of proteins GTF3C4 and RAB1A at lower values (from 0.05 to 0.250), while other alternatives maintain their position. It is also observed that protein GTF3C4 is improving its global priority when the weight of position in the F_t_ (solubility alteration) ranking criterion is increasing.

## 4. Discussion

The development of an AOP requires the identification of the biological responses and causal linkages that are triggered upon perturbation by stressors, including chemical and non-chemical. The first challenge is establishing the mechanistic linkages between the MIE, the intermediate events, and the adverse outcome. Aiming to increase our chemical safety, we observe that even though, over the past decades, significant advances have been done in the development of methodologies for MIEs characterization [6,7] still there are AOPs developed lacking this information. Recently the application of high-throughput methods based on the thermal shift assay has facilitated the identification of a list of protein targets in cell lines and zebrafish embryo model system and the prediction of the mechanisms of action from a single chemical and mixtures [13,14]. In this study, we present the integration of two quantitative methods, PISA and AHP that could be applied to any chemical compound in the future. The model chemical for evaluation purposes, TCDD. The TCDD main target AHR a ligand-activated transcription factor has been heavily studied, on the contrary, other possible targets have attracted less attention [30]. This chemical is classified as a persistent organic pollutant and endocrine disruptor compound and offers a well-known AOP based on the binding and consequently activation of the AHR (AOP 21) before being metabolized [31].

With the focus of this study on the unbiased identification of new protein targets of the chemical, we should clarify that any side effort to increase the size of the studied proteome with the exclusive objective to validate AHR, a well-known target of the chemical, was discharged. Those efforts would have involved the introduction of a pre-fractionation before LC-MS/MS analysis would have enlarged the proteome with a considerably increased time and cost [12]. In this study, the abundance of this receptor in the soluble proteome was not sufficient for its identification and therefore, this target was not within the studied proteome that we discussed in this manuscript. However, another methodological modification essential for the analysis of environmental chemicals that was already introduced in previous works has been maintained [13]. We have previously reported that the identification of targets from hydrophobic chemicals increased in robustness by introducing a step of elimination of the membranous vesicles before the thermal shift assay. It eliminates the risk that the microsomal membranes would partially sequestrate molecules of the chemical in the solution. This is a temperature-dependent process and compromised the maintenance of the stable concentration of chemicals and proteome along the thermal shift assay [13,14]. This methodological workflow could render on average a proteome of 2000 proteins from a single n-LC MS/MS separation that offers a larger proteome coverage for the identification of novel targets of environmental chemicals than the method previously applied [10,32]. Our results showed the identification of new interactions between TCDD and a human hepatic soluble proteome, giving us the chance to unravel novel MIEs, that would have never been studied with a single protein assay. The application of PISA to toxicology requires a tight balance between what is the level of completeness of the cellular proteome that could be achieved in a single experiment without reducing the specificity and sensitivity for target identification.

The classical process for individual target validation involves determining target engagement in vitro or in vivo. Here, the purpose of utilizing orthogonal methods was to confirm the chemical and protein interaction at the structural level. However, this is not an alternative for target validation as it did not include an analysis of the effects of the interaction. Our validation approach areas are a confirmation of the results from the PISA method that are constrained to chemical-protein interactions [12]. The results from the molecular docking showed that targets with higher binding affinities to TCDD also have a higher position in the solubility alteration ranking from PISA, confirming the target capability of the protein targets identified. Furthermore, in the nanoDSF assay, the melting temperature of HSPB1 in the presence of three different concentrations of TCDD shifted from 70.3 °C to 63.4 °C, on average, confirming the binding between this protein and TCDD. 

The results from our methodology are presented as a list of proteins and not just a single unique target. Many of the proteins in the list have not been described as targets in the literature. Therefore, attempts to validate the results imply developing tailored-made strategies. This study aimed to improve our mechanistic understanding of the MIE through target identification. Therefore, after confirming the protein-chemical binding by molecular docking and nanoDSF, the PISA results were analyzed for the first time by MCDM techniques for the prioritization of a single protein target for the AOP framework.

To apply the AHP strategy, eight criteria for selecting the priority protein target were used and the criteria: position in F_t_ (solubility alteration) ranking and position in F_c_ *p*-value ranking obtained the highest weight. Those weight high-weight values relate to the level of importance of evidence that protein-chemical interaction data could provide for an MIE. The molecular confirmation of the MIE by PISA here is therefore key in a bottom-up strategy that starts from the molecular event. However, top-down strategies starting from an observed adverse outcome have been more frequently applied to AOP development [5] based on the difficulties in isolating and identifying molecular interactions [27]. The order of the following five criteria in the priority rank is associated with how an observed adverse outcome of a chemical is relevant from a risk assessment perspective i.e., it corresponds to an accepted protection goal or common apical endpoint in an established regulatory guideline study [2,27]. The criterion number of functional and physical protein associations with other protein targets obtained the last position in the rank because this information could be useful in the implementation of AOP networks and is not directly required for the development of AOPs.

After performing the AHP analysis the protein HSPB1 was selected as the prioritized target for the implementation and development of an AOP. However, if the selection would have been based alone on the 2D PISA assay ranks that are only based on solubility alteration and degree of statistical significance, this target would not occupy the first position of the rank. This result shows the relevance of using an integral, systematic approach, where other aspects of the protein further than chemical binding are included. HSPB1 reported 10 negative effects on cells/organs when its functionality is absent, most of them with regulatory significance. Other alternatives reported a maximum of two negative effects. Furthermore, HSPB1 is associated with 4 pathways while other proteins are associated with a maximum of 1, except for protein RAB1A which also is related to 4 pathways. Protein HSPB1 has no functional and physical protein associations with other protein targets, however, its position in the global priority rank of AHP is not affected since this criterion has the lowest weight.

HSPB1 is a small heat shock protein acting as a molecular chaperone probably of the maintenance of a folding-competent state of denatured proteins [33,34]. Besides, various biological processes, including phosphorylation and axonal transport of neurofilament proteins, are regulated through its molecular chaperone activity [35] and, its loss of function is associated with two neurological diseases, Charcot-Marie-Tooth disease [36], and distal hereditary motor neuronopathy [37]. These findings are in concordance with TCDD-reported neurological toxic effects [38,39,40,41], confirming the potential relevance of protein HSPB1 to be used as an MIE of an AOP after target validation in future studies. The selection of HSPB1 as the prioritized protein target was validated through a sensitivity analysis where minor variations in the criterion position in F_t_ (solubility alteration) ranking were assessed. As a result, minor variations in the ranking of protein GTF3C4 were obtained. These fluctuations are expectable since GTF3C4 has the first position in the PISA assay rank regarding solubility alteration. However, protein HSPB1 maintained the first position at every interval validating the result of the AHP method.

The AHP approach presented here overcomes some limitations in target selections. The parameters included in the semiquantitative analysis are not exclusively based on expert input. Expert knowledge is very valuable, but it is frequently based on reported toxicological studies from only specific targets. For any protein that has not yet been studied as a possible target, the expert knowledge is limited or absent and the risk of underweighting the relevance of the new target is difficult to estimate. This study offers the opportunity to unravel novel MIEs that could be studied and subsequently used to develop AOPs. In the case of TCDD, these new insights are of particular interest since the taxonomic domain of applicability of the well-known AOP where TCDD is identified as a stressor excludes mammals. Meaning that this AOP cannot be applied to humans [31].

Although AOPs are not chemical- or stressor-specific, TCDD has been widely used as a compound of reference in toxicology and risk assessment [42,43,44,45]. Therefore, knowing other MIE/AOP that it triggers is of great significance, especially if they can applicable apply to humans. This linkage is not only valuable as an aid for researchers exploring AOPs that may be relevant to a given stressor but for risk assessment decision-makers evaluating chemicals to enable hazard-based regulation. Additionally, the high throughput unbiased identification of protein targets from all proteins in a studied proteome provides the possibility to fill in missing information of already developed AOPs. Altogether, the integration of the AHP approach to support target selection based on PISA target identification could help decision-makers of risk assessment to get access to policy-relevant scientific data and gain in terms of time and resources.

## 5. Conclusions

We showed that the analysis of chemical-protein interactions by the 2D PISA assay provides an extended list of protein targets and that the AHP technique improves the process of data curation and target selection. Applying the semiquantitative method facilitates the definition of a prioritized target associated with the prediction of an MIE that could support new AOPs. The integration of high-throughput identification of chemical targets by proteomics-based thermal shift methods with target selection by a semiquantitative AHP will reduce biased and knowledge gaps for chemical assessment. We expect that the coupling of those methods could facilitate the applications of AOPs in the chemical risk assessment of novel or alternative chemicals.

## Figures and Tables

**Table 1:** Affinity scores obtained by molecular docking for TCDD and the targets obtained by PISA assay and the well-known target, AHR.

Protein	Vina Score (kcal/mol)
AHR	−6.6
GTCF3C4	−7.3
FAM98B	−6.3
HSPB1	−6.9
RAB1A	−7.2
MTPN	−4.8
KIAA0368	PDB format not available
GRHPR	−6.0
CNPY3	−6.4

**Table 2:** Description of the selected criteria that contribute to the overall goal of selecting by AHP from identified protein targets by 2D PISA assay the priority one for AOPs.

Criteria	Source	Description
Position in F_t_ (solubility alteration) ranking	2D PISA assay	Protein with the highest solubility alteration (the lowest position in the ranking) is more likely to bind the chemical
Position in F_c_ *p*-value ranking	2D PISA assay	Protein with the highest significance (the lowest position in the ranking) for solubility alteration is more likely to bind the chemical
Number of diseases where it is involved	UniProt	Protein with the highest number of diseases where it is involved has more relevance to be included in an AOP
Number of reported negative effects on cells/organs/organisms when their functionality is absent	Literature	Protein with the highest number of reported negative effects on PubMed under the search criteria “lack/absence/knockdown/depletion/knockout of the name of the protein” has more relevance to be included in an AOP
Relevance of reported negative effects on cells/organs/organisms when their functionality is absent	Expertise criteria	Negative effects with regulatory significance (accepted protection goal or common apical endpoint in an established regulatory guideline study) are more relevant
Number of pathways where it has participation	Reactome/ Metabolic Atlas	Protein with the highest number of pathways where it has participation has more relevance to be included in an AOP
Relevance of pathways where it has participation	Expertise criteria	Pathways associated with adverse outcomes are more relevant
Number of functional and physical protein associations with other protein targets	STRING	Protein with the highest number of protein associations with other protein targets has more relevance to be included in an AOP

**Table 3:** Pairwise comparison matrix for the criteria at level 2 of the hierarchy and the computed values of priority vector, λ_max_, CI, RI, and CR.

	Position in F_t_ (Solubility Alteration) Ranking	Position in F_c_ *p*-Value Ranking	Number of Diseases Where It Is Involved	Number of Reported Negative Effects on Cells/Organs/Organisms When Functionality Is Absent	Relevance of Reported Negative Effects on Cells/Organs/Organisms When Functionality Is Absent	Number of Pathways Where It Has Participation	Relevance of Pathways Where It Has Participation	Number of Functional and Physical Protein Associations with Other Protein Targets	Priority Vector
Position in F_t_ (solubility alteration) ranking	1	1	5	5	3	5	3	7	0.258
Position in F_c_ *p*-value ranking	1	1	5	5	3	5	3	7	0.258
Number of diseases where it is involved	1/5	1/5	1	1	1/3	2	1/3	5	0.060
Number of reported negative effects on cells/organs/organisms when functionality is absent	1/5	1/5	1	1	1/5	3	1/5	5	0.061
Relevance of reported negative effects on cells/organs/organisms when functionality is absent	1/3	1/3	3	5	1	5	3	7	0.166
Number of pathways where it has participation	1/5	1/5	1/2	1/3	1/5	1	1/5	5	0.044
Relevance of pathways where it has participation	1/3	1/3	3	5	1/3	5	1	7	0.133
Number of functional and physical protein associations with other protein targets	1/7	1/7	1/5	1/5	1/7	1/5	1/7	1	0.021
λ_max_ = 8.831			CI = 0.119		RI = 1.41		CR = 0.084		

**Table 4:** Local priority for each criterion and global priority of the protein targets (alternatives). The Criteria priority vector is shown in bold.

Protein	Local Priority								Global Priority
	Position in F_t_ (Solubility Alteration) Ranking	Position in F_c_ *p*-Value Ranking	Number of Diseases Where It Is Involved	Number of Reported Negative Effects on Cells/Organs/Organisms When Functionality Is Absent	Relevance of Reported Negative Effects on Cells/Organs/Organisms When Functionality Is Absent	Number of Pathways Where It Has Participation	Relevance of Pathways Where It Has Participation	Number of Functional and Physical Protein Associations with Other Protein Targets	
	0.258	0.258	0.060	0.061	0.166	0.044	0.133	0.021	
**GTF3C4**	0.093	0.130	0.006	0.006	0.016	0.004	0.008	0.002	0.265
**FAM98B**	0.067	0.019	0.006	0.006	0.016	0.004	0.015	0.002	0.135
**HSPB1**	0.048	0.064	0.058	0.052	0.118	0.035	0.037	0.002	0.414
**RAB1A**	0.035	0.047	0.006	0.022	0.062	0.035	0.037	0.019	0.264
**MTPN**	0.024	0.103	0.006	0.006	0.016	0.001	0.002	0.010	0.168
**KIAA0368**	0.016	0.082	0.006	0.011	0.016	0.000	0.001	0.010	0.142
**GRHPR**	0.010	0.011	0.042	0.018	0.149	0.004	0.055	0.002	0.291
**CNPY3**	0.007	0.035	0.042	0.018	0.149	0.004	0.041	0.002	0.297

**Table 5:** The fluctuation in the global priority of the alternatives when minor variations are done to the criterion position in F_t_ (solubility alteration) ranking.

Protein	Global Priority Fluctuation																		
	0.050	0.100	0.150	0.200	0.250	0.300	0.350	0.400	0.450	0.500	0.550	0.600	0.650	0.700	0.750	0.800	0.850	0.900	0.950
**GTF3C4**	0.190	0.208	0.226	0.244	0.262	0.280	0.298	0.316	0.334	0.352	0.370	0.388	0.406	0.424	0.442	0.460	0.478	0.496	0.514
**FAM98B**	0.081	0.094	0.107	0.120	0.133	0.146	0.159	0.172	0.185	0.198	0.211	0.224	0.237	0.249	0.262	0.275	0.288	0.301	0.314
**HSPB1**	0.375	0.385	0.394	0.403	0.413	0.422	0.431	0.441	0.450	0.460	0.469	0.478	0.488	0.497	0.506	0.516	0.525	0.535	0.544
**RAB1A**	0.236	0.242	0.249	0.256	0.263	0.270	0.276	0.283	0.290	0.297	0.303	0.310	0.317	0.324	0.330	0.337	0.344	0.351	0.357
**MTPN**	0.149	0.154	0.158	0.163	0.167	0.172	0.177	0.181	0.186	0.190	0.195	0.200	0.204	0.209	0.213	0.218	0.223	0.227	0.232
**KIAA0368**	0.129	0.132	0.135	0.138	0.141	0.144	0.147	0.150	0.153	0.156	0.159	0.162	0.165	0.168	0.171	0.174	0.178	0.181	0.184
**GRHPR**	0.283	0.285	0.287	0.289	0.291	0.293	0.294	0.296	0.298	0.300	0.302	0.304	0.306	0.308	0.310	0.312	0.314	0.316	0.318
**CNPY3**	0.292	0.293	0.294	0.296	0.297	0.298	0.300	0.301	0.302	0.304	0.305	0.306	0.307	0.309	0.310	0.311	0.313	0.314	0.315

## Data Availability

The mass spectrometry proteomics data have been deposited to the ProteomeXchange Consortium via the PRIDE [23] partner repository with the dataset identifier PXD033056.

## Supplementary Materials

The following supporting information can be downloaded at: https://www.mdpi.com/article/10.3390/toxics11020189/s1, Figure S1: Two dimensions proteome integral solubility alteration assay workflow for chemical target identification. Adapted from Lizano-Fallas et al., 2021; Table S1: Database containing the information of each protein target (alternatives) for each criterion; Table S2: Pairwise comparison matrices for the alternatives at level 3 of the hierarchy for each criterion and the computed values of local priority vector, principal eigenvalue (λ_max_), consistency index (CI), random consistency index (RI), and consistency ratio (CR) for each matrix [36,37,46,47,48,49,50,51,52,53,54,55,56,57].
