# Supplementary material for: Prediction of Molecular Initiating Events for Adverse Outcome Pathways Using High-Throughput Identification of Chemical Targets
Source: Toxics. 2023 Feb 17;11(2):189. doi: 10.3390/toxics11020189 (PMC9965981; doi:10.3390/toxics11020189)
Supplement: Supplementary file 1 [file toxics-11-00189-s001.zip › toxics-2208841-supplementary.pdf]

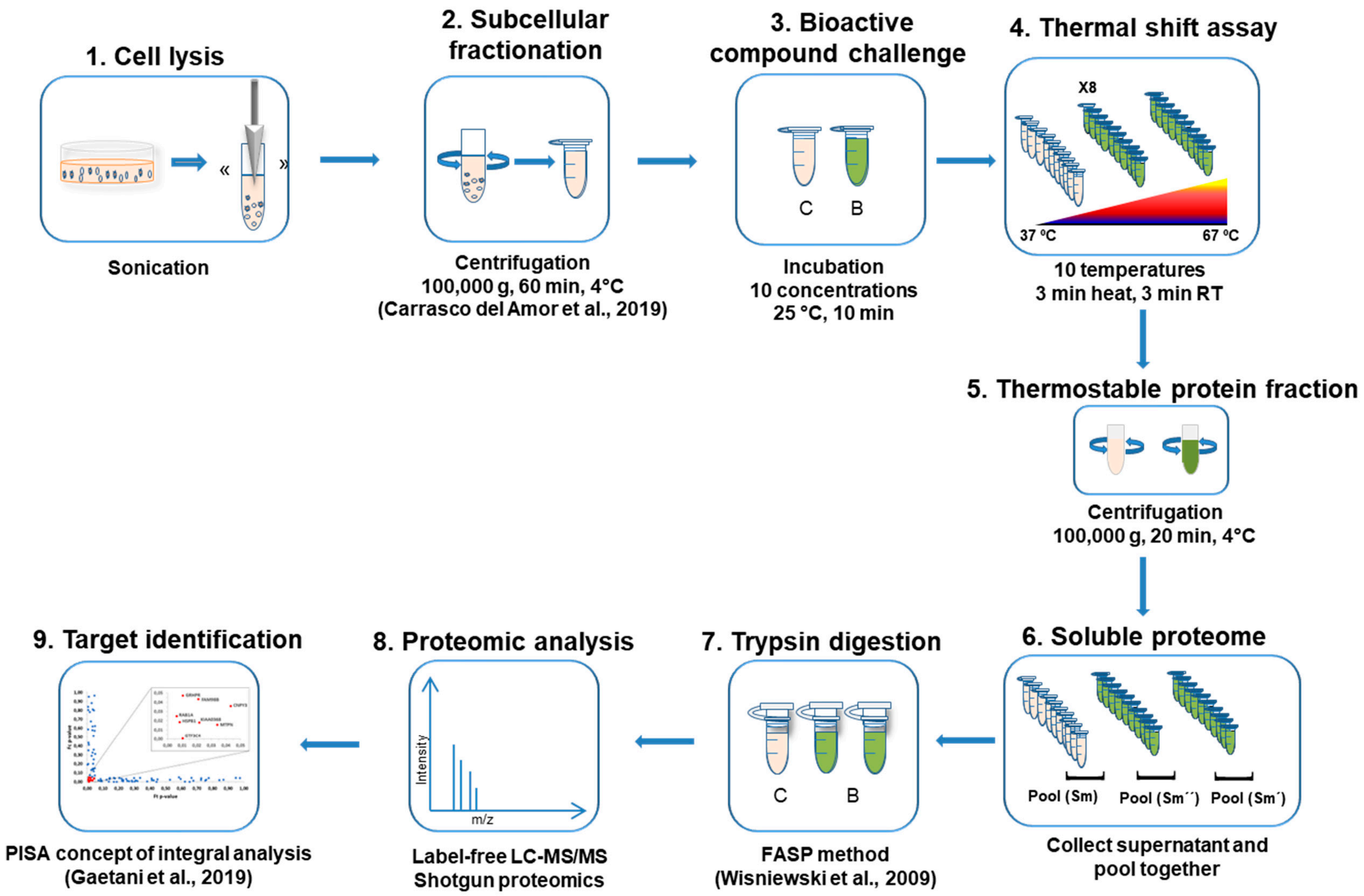

Figure S1. Two dimensions proteome integral solubility alteration assay workflow for chemical target identification. Adapted from Lizano-Fallas et al., 2021.

Table S1. Database containing the information of each protein target (alternatives) for each criterion.

Protein name	Protein name abbreviation	Position in Ft (solubility alteration) ranking	Position in Fc p-value ranking	Number of diseases where it is involved	Number of reported negative effects on cells/organs when functionality is absent	Relevance of reported negative effects on cells/organs when functionality is absent	References	Number of pathways where it has participation	Relevance of pathways where it has participation	Number of functional and physical protein associations with other protein targets
General transcription factor 3C polypeptide 4	GTF3C4	1	1	0	0	NA	NA	1	Gene expression (Transcription): RNA Polymerase III Transcription	0
Protein FAM98B	FAM98B	2	7	0	0	NA	NA	1	Metabolism of RNA: tRNA processing in the nucleus	0
Heat shock protein beta-1	HSPB1	3	4	2	10	Charcot-Marie-Tooth axonal neuropathy; distal hereditary motor neuropathy; myopathy; reduced antioxidant capacity; decreased cell viability and increased the incidence of UVB-induced apoptosis; negative effect on cell growth in chinese hamster ovary cells; attenuated contractile force in human bladder smooth muscle cells; impact on calcium homeostasis and muscle energy	36-37, 46-53	4	Signal transduction: signaling by VEGF, extra-nuclear estrogen signaling and MAPK6/MAPK4 signaling; metabolism of ARN: regulation of mRNA stability by proteins that bind AU-rich elements	0

					metabolism; provokes an accumulation of GATA-1 and impairs terminal maturation in CD34+ human cells; ultrastructural abnormalities in the myofibrillar structure in mouse (e.g. destructured myofibrils and higher gaps between myofibrils)				
Ras-related protein Rab-1A	RAB1A	4	5	0	2	Synthetic lethal in a haploid human cell line (critical for the survival and/or growth of MDCK cells), Golgi stack and ribbon structures disruption, and endoplasmic reticulum (ER)-to-Golgi trafficking inhibition; perinuclear clustering of early endosomes and delayed transferrin recycling in MDCK cells	54-55	4	Vesicle-mediated transport: COPI-mediated anterograde transport, COPII-mediated vesicle transport and intra-Golgi and retrograde Golgi-to-ER traffic; metabolism of proteins: RAB geranylgeranylation	2
Myotrophin	MTPN	5	2	0	0	NA	NA	Protein not found in the sources	NA	1
Proteasome adapter and scaffold protein ECM29	KIAA0368	6	3	0	0	NA	NA	Protein not found in the sources	NA	1

Glyoxylate reductase/hydroxypyruvate reductase	GRHPR	7	8	1	1	Primary hyperoxaluria type II (PH2)	56	1	Metabolism of aminoacids and derivatives: glyoxylate metabolism and glycine degradation	0
Protein canopy homolog 3	CNPY3	8	6	1	1	Early-onset epileptic encephalopathies, including West syndrome	57	1	Immune system: trafficking and processing of endosomal TLR	0

Table S2. Pairwise comparison matrices for the alternatives at level 3 of the hierarchy for each criterion and the computed values of local priority vector, principal eigenvalue (λ_{max}), consistency index (CI), random consistency index (RI), and consistency ratio (CR) for each matrix.

**Position in Ft (solubility alteration) ranking**	GTF3C4	FAM98B	HSPB1	RAB1A	MTPN	KIAA0368	GRHPR	CNPY3	Priority vector
GTF3C4	1	2	3	4	5	6	7	8	0.360
FAM98B	1/2	1	2	3	4	5	6	7	0.259
HSPB1	1/3	1/2	1	2	3	4	5	6	0.188
RAB1A	1/4	1/3	1/2	1	2	3	4	5	0.135
MTPN	1/5	1/4	1/3	1/2	1	2	3	4	0.092
KIAA0368	1/6	1/5	1/4	1/3	1/2	1	2	3	0.061
GRHPR	1/7	1/6	1/5	1/4	1/3	1/2	1	2	0.039
CNPY3	1/8	1/7	1/6	1/5	1/4	1/3	1/2	1	0.026
	λmax =	9.563	CI =	0.223	RI=	1.41	CR =	0.158	

**Position in Fc p-value ranking**	GTF3C4	FAM98B	HSPB1	RAB1A	MTPN	KIAA0368	GRHPR	CNPY3	Priority vector
GTF3C4	1	7	4	5	2	3	8	6	0.502
FAM98B	1/7	1	1/4	1/3	1/6	1/5	2	1/2	0.073
HSPB1	1/4	4	1	2	1/3	1/2	5	3	0.247
RAB1A	1/5	3	1/2	1	1/4	1/3	4	2	0.183
MTPN	1/2	6	3	4	1	2	7	5	0.400
KIAA0368	1/3	5	2	3	1/2	1	6	4	0.318
GRHPR	1/8	1/2	1/5	1/4	1/7	1/6	1	1/3	0.041
CNPY3	1/6	2	1/3	2	1/5	1/4	3	1	0.134
	λmax =	8.551	CI =	0.079	RI=	1.41	CR =	0.056	

**Number of diseases where it is involved**	GTF3C4	FAM98B	HSPB1	RAB1A	MTPN	KIAA0368	GRHPR	CNPY3	Priority vector
GTF3C4	1	1	1/9	1	1	1	1/7	1/7	0.102
FAM98B	1	1	1/9	1	1	1	1/7	1/7	0.102

**Number of reported negative effects on cells/organs/organisms when functionality is absent**	GTF3C4	FAM98B	HSPB1	RAB1A	MTPN	KIAA0368	GRHPR	CNPY3	Priority vector
GTF3C4	1	1	1/9	1/5	1	1	1/3	1/3	0.100
FAM98B	1	1	1/9	1/5	1	1	1/3	1/3	0.100

HSPB1	9	9	1	9	9	9	5	5	0.968
RAB1A	1	1	1/9	1	1	1	1/7	1/7	0.102
MTPN	1	1	1/9	1	1	1	1/7	1/7	0.102
KIAA0368	1	1	1/9	1	1	1	1/7	1/7	0.102
GRHPR	7	7	1/5	7	7	7	1	1	0.708
CNPY3	7	7	1/5	7	7	7	1	1	0.708
	λmax =	8.042	CI =	0.006	RI=	1.41	CR =	0.004	

HSPB1	9	9	1	7	9	9	8	8	0.853
RAB1A	5	5	1/7	1	5	5	3	3	0.369
MTPN	1	1	1/9	1/5	1	1	1/3	1/3	0.100
KIAA0368	1	1	1/9	1/5	1	1	1/3	1/3	0.173
GRHPR	3	3	1/8	1/3	3	3	1	1	0.297
CNPY3	3	3	1/8	1/3	3	3	1	1	0.297
	λmax =	8.166	CI =	0.024	RI=	1.41	CR =	0.017	

**Relevance of reported negative effects on cells/organs/organisms when functionality is absent**	GTF3C4	FAM98B	HSPB1	RAB1A	MTPN	KIAA0368	GRHPR	CNPY3	Priority vector
GTF3C4	1	1	1/9	1/5	1	1	1/9	1/9	0.097
FAM98B	1	1	1/9	1/5	1	1	1/9	1/9	0.097
HSPB1	9	9	1	1/7	9	9	1	1	0.715
RAB1A	5	5	7	1	5	5	1/7	1/7	0.374
MTPN	1	1	1/9	1/5	1	1	1/9	1/9	0.097
KIAA0368	1	1	1/9	1/5	1	1	1/9	1/9	0.097
GRHPR	9	9	1	7	9	9	1	1	0.900
CNPY3	9	9	1	7	9	9	1	1	0.900

**Number of pathways where it has participation**	GTF3C4	FAM98B	HSPB1	RAB1A	MTPN	KIAA0368	GRHPR	CNPY3	Priority vector
GTF3C4	1	1	1/9	1/9	0	0	1	1	0.089
FAM98B	1	1	1/9	1/9	0	0	1	1	0.089
HSPB1	9	9	1	1	0	0	9	9	0.801
RAB1A	9	9	1	1	0	0	9	9	0.801
MTPN	0	0	0	0	1	0	0	0	0.015
KIAA0368	0	0	0	0	0	1	0	0	0.005
GRHPR	1	1	1/9	1/9	0	0	1	1	0.089
CNPY3	1	1	1/9	1/9	0	0	1	1	0.089

	λmax =	8.496	CI =	0.071	RI=	1.41	CR =	0.050			λmax =	8.274	CI =	0.039	RI=	1.41	CR =	0.028	
**Relevance of pathways where it has participation**	GTF3C4	FAM98B	HSPB1	RAB1A	MTPN	KIAA0368	GRHPR	CNPY3	Priority vector	**Number of functional and physical protein associations with other protein targets**	GTF3C4	FAM98B	HSPB1	RAB1A	MTPN	KIAA0368	GRHPR	CNPY3	Priority vector
GTF3C4	1	1/2	1/3	1/3	0	0	1/5	1/3	0.062	GTF3C4	1	1	1	1/9	1/5	1/5	1	1	0.099
FAM98B	2	1	1/4	1/4	0	0	1/5	1/4	0.116	FAM98B	1	1	1	1/9	1/5	1/5	1	1	0.099
HSPB1	3	4	1	1	0	0	1/3	1	0.276	HSPB1	1	1	1	1/9	1/5	1/5	1	1	0.099
RAB1A	3	4	1	1	0	0	1/3	1	0.276	RAB1A	9	9	9	1	4	4	9	9	0.935
MTPN	0	0	0	0	1	0	0	0	0.015	MTPN	5	5	5	1/4	1	1	5	5	0.493
KIAA0368	0	0	0	0	0	1	0	0	0.005	KIAA0368	5	5	5	1/4	1	1	5	5	0.493
GRHPR	5	5	3	3	0	0	1	1/3	0.416	GRHPR	1	1	1	1/9	1/5	1/5	1	1	0.099
CNPY3	3	4	1	1	0	0	3	1	0.306	CNPY3	1	1	1	1/9	1/5	1/5	1	1	0.099
	λmax =	8.038	CI =	0.005	RI=	1.41	CR =	0.004			λmax =	8.000	CI =	0.000	RI=	1.41	CR =	0.000	
